# Supplementary material for: An ecological approach to structural flexibility in online communication systems
Source: Nat Commun. 2021 Mar 29;12:1941. doi: 10.1038/s41467-021-22184-2 (PMC8007599; doi:10.1038/s41467-021-22184-2)
Supplement: Supplementary file 1 — Supplementary Information [file 41467_2021_22184_MOESM1_ESM.pdf]

Supplementary Information

# An Ecological Approach to Structural Flexibility in Online Communication Systems

María J. Palazzi[1], Albert Solé-Ribalta[1,2], Sandro Meloni[3], Violeta Calleja-Solanas[3], Carlos A. Plata[4,5], Samir Suweis[4] and Javier Borge-Hotlhoefer[1]

[1]Internet Interdisciplinary Institute (IN3), Universitat Oberta de Catalunya, Barcelona, Catalonia, Spain

[2]URPP Social Networks, University of Zurich, Zurich, Switzerland

[3]IFISC, Institute for Cross-Disciplinary Physics and Complex Systems (CSIC-UIB), 07122, Palma de Mallorca, Spain

[4]Dipartimento di Fisica e Astronomia G. Galilei, Università di Padova, Via Marzolo 8, Padova, 35131, Italy.

[5]Université Paris-Saclay, CNRS, LPTMS, 91405 Orsay, France.

February 22, 2021

# Supplementary note 1: Empirical analysis

## Datasets

The empirical data employed in this work was collected from the online platform `www.twitter.com`. Following the analogy with interactions in ecological systems, two types of species are considered: users and hashtags (memes). For each tweet on the different datasets, we only extracted the user's name, the hashtags in the tweets, and the time at which they were posted.

We considered six events of different nature: the Spanish general elections april 2019 (28A), the 2015 Nepal earthquakes, the 2012 UEFA European Football Championship, the 2014 Catalan self-determination referendum, the 2015 Charlie Hebdo Shooting and the 2014 Hong Kong streets protests. All the datasets excepting the ones from the Spanish general elections and the Catalan self-determination referendum were collected by Zubiaga A. in [24]. In the following, we report details concerning these events and the associated Twitter data sets.

1. Spanish general Elections (April 2019): The April 2019 Spanish general elections were held on Sunday, 28 April 2019, to elect the 13th bicameral legislative chambers of the Kingdom of Spain, the 350 seats in the Congress of Deputies and 208 out of 266 seats in the Senate. The observation period started at the beginning of the electoral campaign, on the 12th of April and lasted until the 6th of May, a few days before the beginning of the electoral campaign for the election of the 54 Spanish members of the European Parliament. Hence, the observation period was marked by intense political activity. For this event, we collected a dataset composed of $3,0107,629$ unique tweets containing at least one hashtag, with a total $124,062$ unique hashtags and $1,883,468$ users. The dataset was collected by selecting all the tweets containing at least one of a total set composed of 300 relevant keywords that could be either user names or hashtags related to the electoral process, i.e, names of candidates, electoral actvities (debates, meetings) and name of the parties involved, etc. The collection of this dataset was carried out in collaboration with Joan Tomàs Matamalas.

2. Nepal Earthquake (April-May 2015): The next dataset taken into consideration for analysis corresponds to an unexpected event, specifically, a series of earthquakes registered in Nepal in 2015. The first earthquake occurred on the 25 of April 2015, registering around 9000 casualties. This event was followed by several continued aftershocks, with a major aftershock of similar magnitude of the first quake, registered on May 12th. Given the unpredictable nature of this type of event, we have focused on the study of the second major earthquake. The observation period covers a total of six days, from 8 to 14 of May, a few days after the second aftershock. The dataset contains $1,918,045$ unique tweets containing at least one hashtag, with a total $35,795$ unique hashtags and $810,744$ users. The dataset was collected by selecting all the tweets containing at least one of the following hashtags or keywords: *nepal*, *earthquake*, *#nepalearthquake*.

3. Catalan self-determination referendum (Nov 2014): The second event corresponds to the Citizen's Participation Process on the Political Future of Catalonia, a popular consultation about the process of independence of Catalonia from the Spanish Kingdom. The consultation was held on Sunday, 9 November 2014, after the approval decree was signed by the president of Catalonia on September 27 of the same year. The dataset contains $220,364$ unique tweets containing at least one hashtag, with a total $18,116$ unique hashtags and $78,270$ users, ranging from September 1st to November 13 of 2014. In a similar manner to the Spanish election dataset, this dataset was collected by selecting all the tweets containing at least one of a preselected set of $\approx 70$ hashtags and $\approx 50$ Twitter accounts related to the referendum process and the Catalan independence movement.

4. European football championship (2012): In third place, we considered the 2012 UEFA Football Championship, an European championship for men's national football teams. The tournament was held between 2 June and 1 July of 2012, and co-hosted by Poland and Ukraine. The observation period started a day before of the quarter-finals, on 19 June and lasted until the 4th of July, right after the final game. It contains $3,907,418$ unique tweets containing at least one hashtag, with a total $147,646$ unique hashtags and $1,325,631$ users. This dataset was collected by selecting all the tweets containing the hashtag *#euro2012*.

5. Hong Kong protests (Sept-Oct 2014): An additional dataset taken into consideration corresponds to a series of streets protests that took place in Hong Kong from September to December 2014. The protests, are often referred to as the Umbrella Movement or Occupy movement. The protests were initiated after a proposal from the Standing Committee of the National People's Congress to reform the electoral law. The dataset contains $826,194$ unique tweets containing at least one hashtag, with a total $30,105$ unique hashtags and $239,432$ users. The observation period started on 27th of September, right after the protests escalated, resulting in several people detained, until October 10. The dataset was collected by selecting all the tweets containing at least one of the following hashtags or keywords: *#hongkong*, *#umbrellamovement*, *#occupycentral*, *#hongkongprotests*, *#occupyhongkong*.

6. Charlie Hebdo Shooting (Jan 2015): The last dataset taken into consideration for analysis also corresponds to an unexpected event, specifically, the shooting perpetrated at the offices of the french magazine Charlie Hebdo, on January 7 2015. On the morning of January 7 of 2015, two heavily armed brothers forced their entry into the magazine offices, killing 12 people and injuring 11 more. The dataset contains $6,002,087$ unique tweets containing at least one hashtag, with a total $102,799$ unique hashtags and $2,001,826$ users. The observation period started on the 8th of January, right after the shooting took place and lasted until the 10th of January, after the two main suspects were killed. The dataset was collected by selecting all the tweets containing at least one of the following hashtags or keywords: *#jesuischarlie*, *#charliehebdo*, *charlie hebdo paris*.

Supplementary Table 1: Summary of our datasets.

| Dataset | Data length | Total days | Tweets | Users | Hashtags |
|---|---|---|---|---|---|
| 2019 Spanish general elections | April 12 - May 6 | 24 | 30,107,629 | 1,883,468 | 124,062 |
| 2015 Nepal Earthquake | May 8-14 | 6 | 1,918,045 | 810,744 | 35,795 |
| 2014 Catalan referendum | Sep 2 - Nov 12 | 10 | 220,364 | 78,270 | 18,116 |
| 2012 UEFA football championship | Jun 19 - July 4 | 15 | 3,907,418 | 1,325,631 | 147,646 |
| 2014 Hong Kong Protests | Sep 27 - Oct 7 | 10 | 826,194 | 239,432 | 30,105 |
| 2015 Charlie Hebdo shooting | Jan 8-9 | 2 | 6,002,087 | 2,001,826 | 102,799 |

## Matrix construction

As it is explained in the main text, we attempt to account for how the interactions between users and hashtags change over time. Prior the construction of the interaction matrices, we performed a selection criteria that allowed us to capture the structural changes of the data in a smooth way, and, at the same time, reducing the computational cost.

For each dataset, we split the timestream into chunks according to non-overlapping time windows with three hours of duration $\omega = 3$h, Supplementary Fig. 1a. For each chunk, we built matrices $a_{uh}^{(t)}$ containing the $N_U = 2000$ most active unique users and a variable number of hashtags $N_H$, depending on the amount produced by those 2000 users [8], Supplementary Fig. 1b. Each cell in the matrices $a_{uh}^{(t)}$ is equal to 1 if user $u$ has posted a message containing the hashtag $h$ at least once, and 0 otherwise. Note that each matrix will have a different duration, spanning from a few minutes during the times of high activity (when an event is taking place), to the total duration of the time window. For each one of these 3-hour chunks, we select the matrices that are closer to the middle of the time window, e.g., $a^{(t\approx\omega/2)}$ to perform the structural analysis Supplementary Fig. 1c. Around the periods of high activity –on the onset of the events– the procedure is repeated considering time windows of 15 minutes of duration.

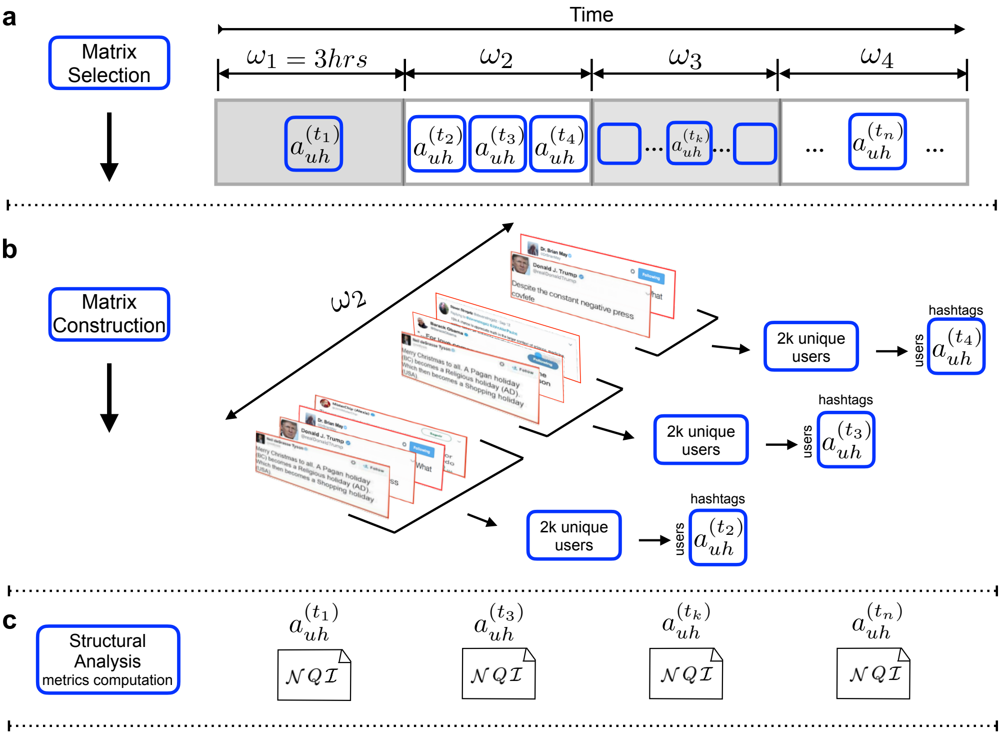


Supplementary Figure 1: **Schematic representation of the implemented methodology in the analysis of empirical data.** The applied methodology comprises three steps: Selection and construction of the adjacency matrices (panels **a** and **b**, respectively). Structural analysis of the selected matrices by means of nestedness, modularity and in-block nestedness (panel **c**).

It is also important to highlight that the $a_{uh}^{(t)}$ matrices may not contain the same nodes across $t$: as time advances, users join (disappear) as they start (cease) to show activity; the same applies for hashtags, which might or might not be in the focus of attention of users. In the following, we quantify the temporal continuity of users and hashtags over time ('species turnover'). On the hashtags side, Supplementary Figure 2a represents the overlap between consecutive hashtag coversets ($|H_t \cap H_{t-1}|/\min(|H_t|, |H_{t-1}|)$), showing a strong persistence of the main hashtags in general, and above 60% during the highlighted events (the trend is smoothed to remove the fluctuations during the night periods, in which activity remains very low; the raw plot reaches a maximum at $\sim$80%, and a minimum at $\sim$20%).

Supplementary Figure 2b shows, conversely, the overlap between consecutive user coversets (computed as $|U_t \cap U_{t-1}|/\min(|U_t|, |U_{t-1}|)$), which remains relatively constant and low between 10 and 15% (again, the trend is smoothed). This volatile situation (both for users and hashtags) is quite normal in time-resolved ecology field studies [2, 19, 11], where the emphasis is placed on the system's dynamics rather than individual species. In our case, despite the composition of users, hashtags, and their interactions may vary across minutes, hours, and days, evidence suggests that networks are composed of a reliable core of generalist species, accompanied by a changing suite of specialist species [23].

## Structural measures over the empirical matrices

We explore the structural evolution of the network by means of three arrangements: nestedness [18, 4] at the macroscale (Supplementary Fig. 3a), and modularity [16, 5] (Supplementary Fig. 3b) at the mesoscale. Further-

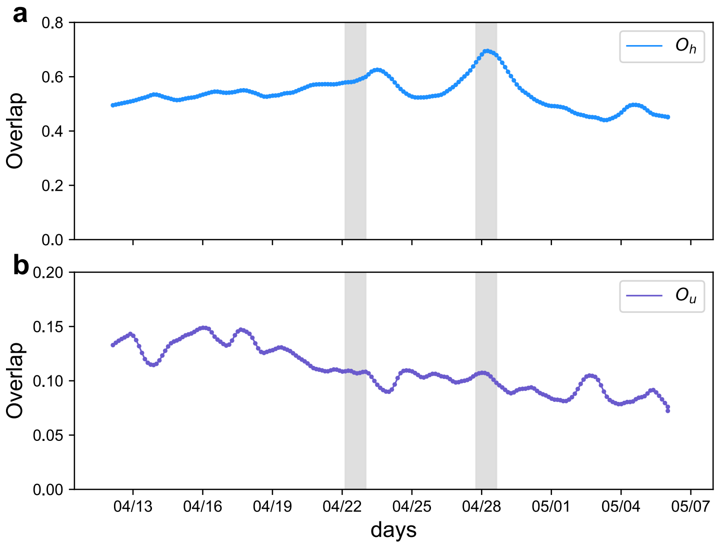


Supplementary Figure 2: **Species turnover.** Overlap across time between the most relevant hashtags (**a**) and users (**b**) in consecutive snapshots of the Spanish dataset.

more, we will also consider in-block nested patterns that consist on the combination of nestedness and modularity in the same network, i.e., communities with a nested structure within [15], Supplementary Fig. 3c. The explicit definitions of nestedness, modularity and in-block nestedness were already provided in the main text.

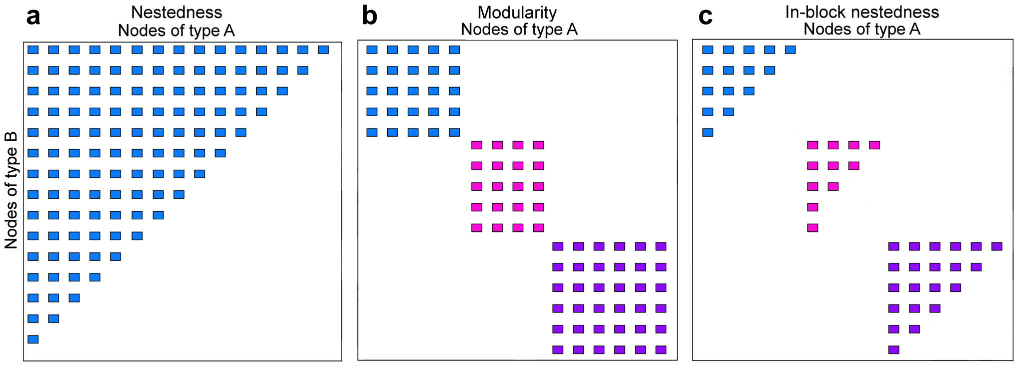


Supplementary Figure 3: **Idealised examples of the structural organisation studied in the paper.** **a** shows a perfectly nested organisation. **b** the adjacency matrix of modular network and **c** an idealised network with in-block nested structure.

## Structural flexibility in information systems: additional datasets

In this section we present four additional results corresponding to different portions of Twitter activity. For the sake of consistency, once again, we analyse these datasets monitoring the system's modularity [16] ($Q$), nestedness [18, 4, 6] ($\mathcal{N}$) and in-block nestedness ($\mathcal{I}$) [20].

Supplementary Figure 4 shows the evolution of $Q$ and $\mathcal{N}$ for the Catalan self-determination referendum from

2014 (panel a), the 2012 European football championship (panel b), the 2014 Hong Kong street protests (panel c) and the 2015 Charlie Hebdo Magazine shooting (panel d). The duration of the snapshot was adjusted to provide a better visualization of the structural transitions during the different events, and highlighted the location of the events in each plot. Some of these events are pointed out in the pair of insets in each figure.

Overall, we observe that for all different datasets the behaviour is in qualitative agreement with the ones presented at the main text. First, the anticorrelated behaviour between global nestedness and modularity is preserved. Further, in each case, regardless the nature of the different datasets, we observe a smooth transition into self-similar nested arrangements, which develop in accordance to the level of fragmentation of the surrounding conditions, i.e this transition is linked to external events (second row in all panels). The different datasets, regardless of their nature, lie along the lines of the different classes of collective attention described in Lehmann *et al.* [14]. The highly fluctuating pattern in Supplementary Fig. 4(b), corresponding to the UEFA championship, is due to the periodicity in which football games happen throughout the competition, with a slowdown by the end of the period when only the semifinal and final game are left.

Although mentioned in the Datasets Section of this Supplementary Note, it worth highlighting that different data acquisition procedures employed to build the analysed datasets. The Spanish elections and Catalan referendum datasets were collected from a rich collection of hashtags and keywords that were manually chosen following the evolution of the event, even introducing new hashtags –or keywords– as the event unfolded. In contrast, the rest of the datasets were collected from a small set of hashtags (often just one) [24], resulting in the presence of "super"-generalist memes during all the stages of the discussion. Regardless of the possible biases induced by the presence of these "super"-generalist memes in some of the datasets, many (possibly most) important hashtags emerging at later stages are captured as well, since they tend to co-occur with the original chosen keyword. Thus, we were able to capture the different states of collective attention, from fragmented to global stages of public consensus.

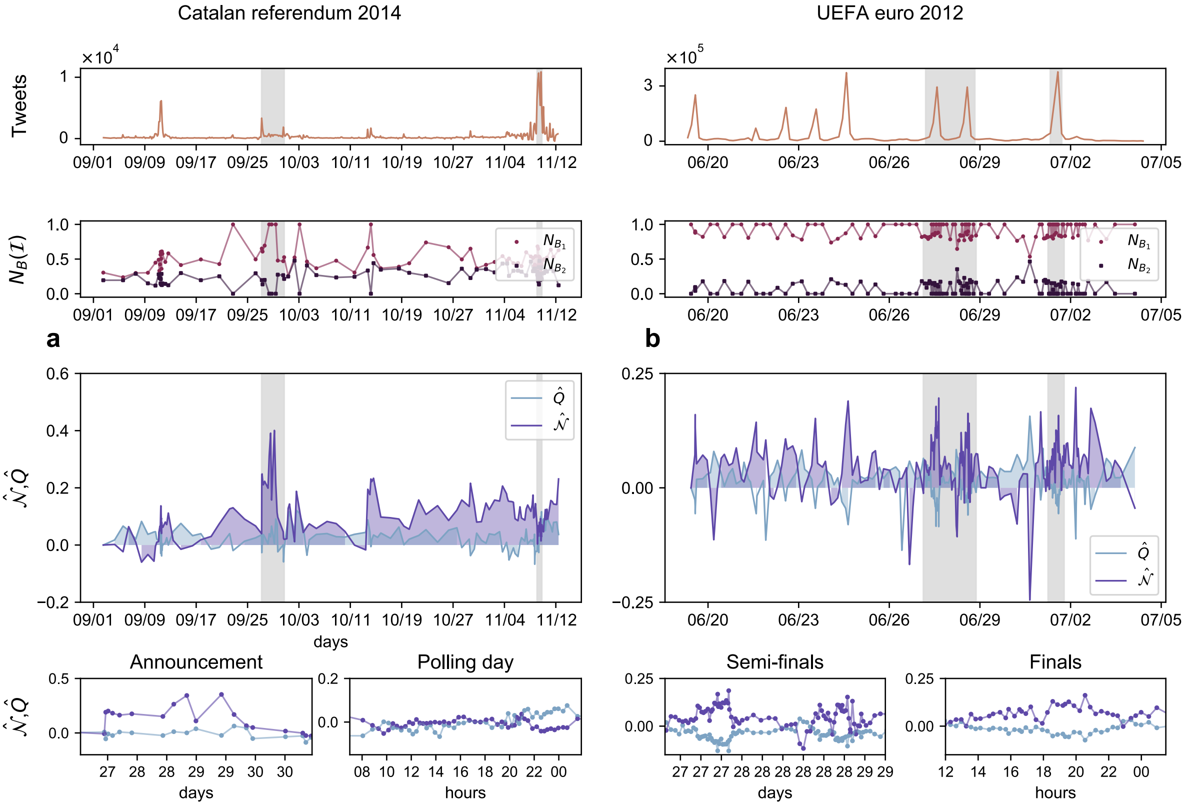

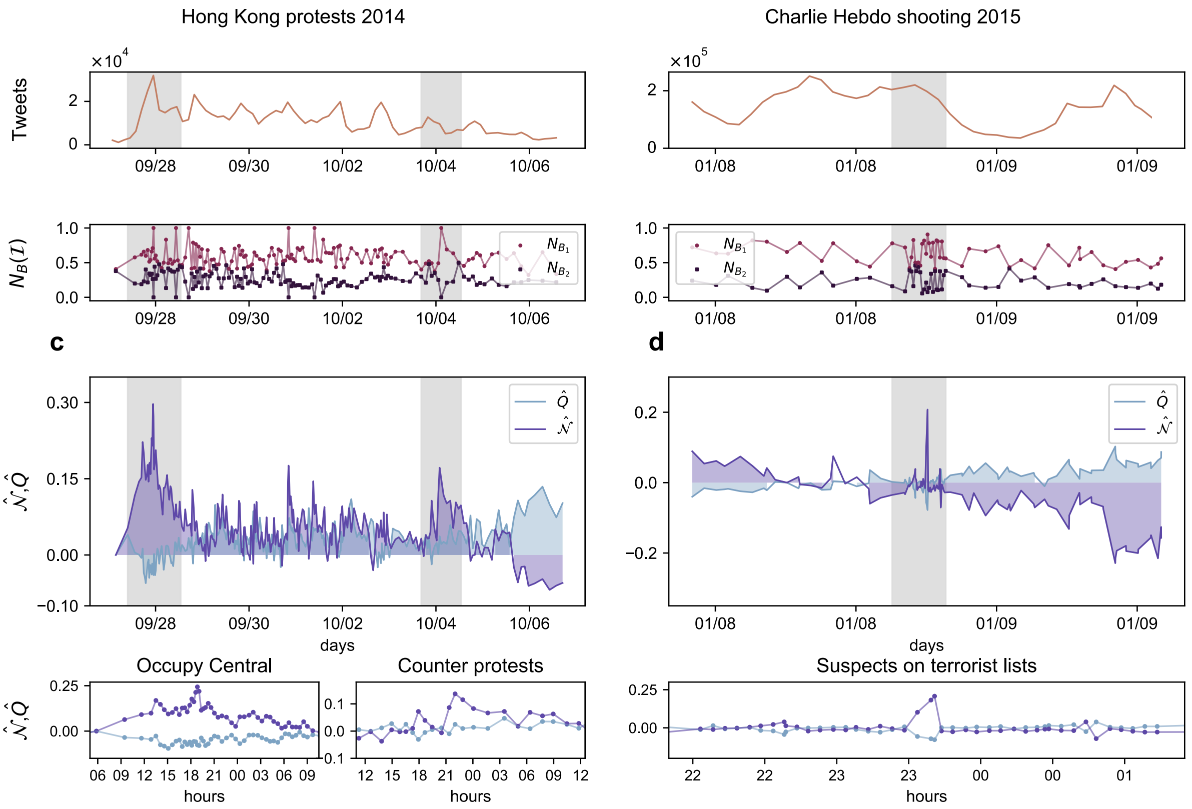


Supplementary Figure 4: **Structural measures over time for four different datasets.** Panel (a) corresponds to the Catalan self-determination referendum held in 2014, panel (b) corresponds to the 2012 UEFA Football Championship, panel (c) corresponds to a series of streets protests ocurred in Hong Kong in 2014. Finally, panel (d) correspond to the Charlie Hebbdo shooting on 2015. In accordance with empirical results presented in the main text, here we observe how a block organisation dominates the system, reflecting the separate interests of users, until external events induce large-scale attention shifts, which rearrange completely the observed architecture towards a macroscale nested pattern. Once again, we highlight specific time windows in each dataset with some identifiable event happening in them.

## Qualitative and quantitative nestedness

Throughout the main text and this Supplementary Information, nestedness is measured from an unweighted (binary) matrix. However, as pointed out in several works (e.g. Blüthgen et al. [7] or Staniczenko et al. [21]), other measures might be needed to discriminate between qualitative from quantitative nestedness, i.e. one that takes into account the distribution of weights in the interaction matrix. As exemplified in [21], an anti-nested pattern can underlie an apparently nested one, when the strongest weights in the network are devoted to the specialist nodes (i.e., those with lower degree).

For the sake of completeness, a, b and c in Supplementary Figure 5 show two user-hashtag matrices, which correspond to the Spanish dataset in two different moments. In one of them, the predominant pattern is in-block nested (a); in the other (b), the predominant pattern is purely nested. The matrices have been arranged to facilitate the visualisation of the corresponding architecture, and the cells are coloured according to the link weight (i.e. how many times user $u$ tweeted hashtag $h$). Following the arguments in Staniczenko et al. [21]), these matrices provide

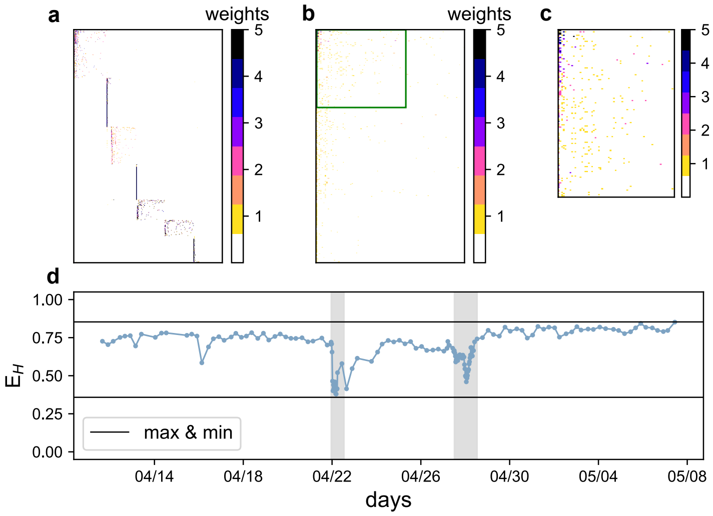


Supplementary Figure 5: **Quantitative nestedness and evenness.** weighted in-block nested (**a**) and nested (**b**) matrices, in which darker colours correspond to stronger links. **c** corresponds to the subset of most generalist users/hashtags in **b**, to highlight the stronger weights in the upper-left part. If only visually, it is apparent that the matrices are qualitatively and quantitatively in-block nested and nested, respectively. **d**: evolution of evenness for the Spanish dataset. As expected, evenness remains relatively high (baseline behaviour), with abrupt dips whenever external shocks affect the system.

a visual insight on whether the qualitatively observed nested patterns are also quantitatively matched. Indeed, one can observe that the strongest links (darker colours) correspond to those among generalists. To better appreciate this pattern in the case of global nestedness, in Supplementary Figure 5c, we draw on the right a submatrix for the first 150 users (and corresponding hashtags).

Finally Supplementary Figure 5 d, shows the evolution of evenness as defined in [7]:

$$E_H = \frac{\sum_h^{N_H} p_h \ln p_h}{\ln N_H} \tag{1}$$

(for the case of hashtags; equivalently for users, just switching $h$ and $N_H$ by $u$ and $N_U$, respectively). As expected, evenness portrays a similar evolution to those of nestedness and modularity: it remains relatively stable in all the period, except for exceptional episodes that disrupt the attention focus in the system.

## Representativeness and robustness of $N_U$

The large size of our several datasets severely handicaps the data processing, and makes the calculations time-consuming (or even prohibitive). As explained in Section  above, we have applied some restrictions to the number of users considered to build the network in each time snapshot, ($N_U = 2000$). Although this may appear as an arbitrary decision, in this section we show that $N_U = 2000$ users (and the number of hashtags produced by those, which varies from slice to slice) are a good representative of the overall activity and of the structural patterns that emerge.

By selecting top contributors (and their associated hashtags), we guarantee that both generalists and specialists will show up –if any nested pattern is to be found. Also, the chances of obtaining a connected matrix are higher (note that the appearance of disconnected components would render a trivially modular network).

In Supplementary Fig. 6(a), we show, for the Spanish dataset, the corresponding proportion of the 2000 unique users, with respect to the total number of users for each snapshot. That is: if the 3−hour window at time $t$ has $N_t$ users, the panel is showing $N_U/N_t$. Clearly, 2000 users represent in general a minority of all the participants –with an average of 40%. In Supplementary Fig. 6 (b), we show the proportion of tweets for which those 2000 users are responsible $(Tw_U/Tw_t)$. It turns out that, for each 3−hour window, these top contributors often account for more than 60% of the overall activity, with an average around 57%. Note that, to avoid the heavy circadian fluctuations (at night, $N_U = 2000$ users represent 100% of the total), both panels are shown as the average on a sliding window scheme.

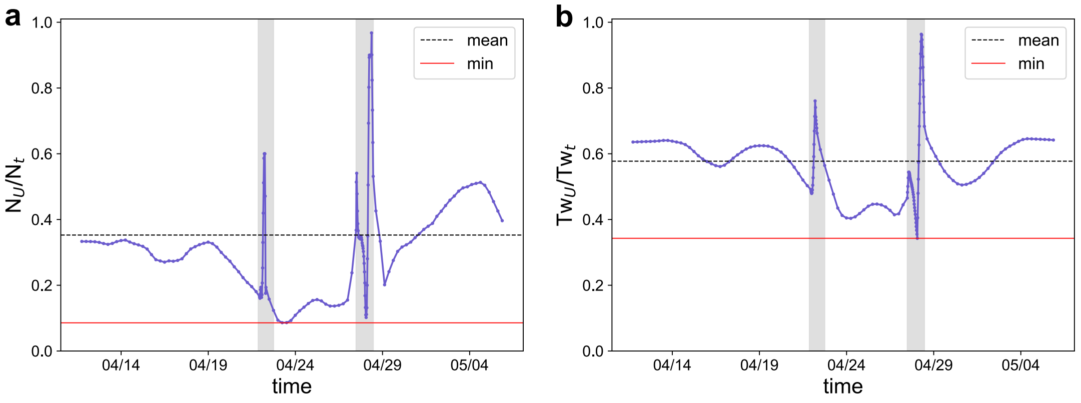


Supplementary Figure 6: **Representativeness of $N_U$. a** Fraction of users $N_U/N_t$ (where $N_t$ is the total number of users in a window at time $t$), which shows that $N_U = 2000$ represents, most of the time, a minority of the total. However, their status as top contributors grants that most of the activity for a given window is captured by that minority **b**.

On the other hand, one might also wonder to what extent the results we present are robust against other network sizes. We have experimented with different thresholds, i.e. $N_U = [500, 1000]$ in the case of the Spanish dataset. For these numbers of users, the evolution of $Q$ and $\mathcal{N}$ was generally indistinguishable from the one reported in Fig. 1 of the main text, see Supplementary Figure 7 below for $N_U = 500$.

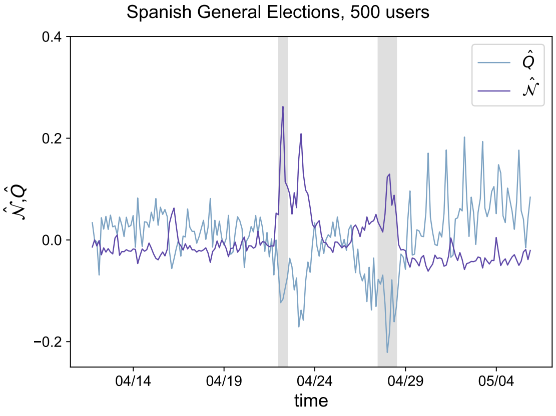


Supplementary Figure 7: $Q$ **and** $\mathcal{N}$ **evolution for the Spanish dataset, with** $N_U = 500$ **users.** As in Figure 1 of the main text, values are measured for 3−hour snapshots. Note that the values for $Q$ and $\mathcal{N}$ are shifted to the initial one.

## Supplementary note 2: Dynamical model and numerical results

As mentioned in the main text, to fully identify the mechanisms behind the observed structural transitions in the empirical data, we have developed an ecology-inspired theoretical model that incorporates four different drivers: competition, mutualism, co-adaptation, and environmental conditions. A detailed description of the model is presented below.

### Niche dynamics

The model is developed for a bipartite network that contains interacting "species" in two classes or guilds (denoted $U$ and $H$, in analogy with users and hashtags). For each species $i$ we assign a niche profile, which is formulated as a Gaussian function $G_i(s)$ with width $\sigma_i$ – for simplicity, we assign the same niche width to all the species– and its center position $\overline{s}_i$ are chosen selected within the interval $[0, 1]$ on a niche axis, with fixed boundary conditions. In the original formulation, the center positions of the niche profiles were randomly distributed along the niche axis. In this work, we assume that each user and hashtag is involved in niche relations according to specified topics of its interest. In particular, a number of $T$ topics are created equidistant on the niche axis. The niche center of the $N_U$ users and $N_H$ hashtags are set in the vicinity of each topic.

### Mutualistic and competitive interactions.

The species are involved in cross-guild mutualistic interactions and into competitive interactions with all the nodes in its own guild proportional to the niche overlap. We define the niche overlap $G_{ij}$ of a pair of nodes $i$ and $j$ as:

$$G_{ij}^{gg'} = \int G_i^g(s) G_j^{g'}(s) ds \tag{2}$$

with $g$ and $g'$ denoting the guild of the considered species, either users or hashtags.

Following this, we define the mutualistic interaction matrix as:

$$\text{Mutualism: } \gamma_{ik}^{UH} = \Omega_m \cdot \theta_{ik} \cdot G_{ik}^{UH} \tag{3}$$

where $\theta_{ik}$ is the adjacency matrix, with entries equal to 1 if $i$ and $k$ interact, and 0 otherwise, and $\Omega_m$ is the intensity coefficients for the mutualistic interaction.

Regarding the competitive interactions, in the context of information ecosystems we distinguish two levels of competition. At the local level, users (hashtags) in the same topic compete to gain visibility among those with related interests (meaning). At the aggregate level, a given topic strives to prevail among other topics. In order to capture this double competition as a trade off between both tendencies in our model, we need to redefine the competitive interaction matrix as:

$$\text{Competition: } \beta_{ij}^{U} = \begin{cases} 1 & \text{if } i = j \\ \Omega_c \left[ \lambda(1 - G_{ij}^{UU}) + (1 - \lambda) G_{ij}^{UU} \right], & \text{otherwise,} \end{cases} \tag{4}$$

where $\Omega_c$ is the intensity coefficients for the competitive interaction and $\lambda \in [0, 1]$ is the inter-intra topic competition parameter, the same definition applies to the competitive interactions among hashtags. For the case $\lambda = 1$, the competitive matrix neglects the competition among users belonging to the same topic. The case when $\lambda = 0$ corresponds to the original formulation [9].

### Population dynamics

The species abundances evolve according to a set of Lotka-Volterra equations with Holling-Type II mutualistic functional response with handling time $h$:

$$\frac{dn_i^U}{dt} = n_i^U \left( \rho_i^U - \sum_j \beta_{ij}^U n_j^U + \frac{\sum_k \gamma_{ik}^{UH} n_k^H}{1 + h \sum_k \theta_{ik}^{UH} n_k^H} \right)$$

$$\frac{dn_i^H}{dt} = n_i^H \left( \rho_i^H - \sum_j \beta_{ij}^H n_j^H + \frac{\sum_k \gamma_{ik}^{HU} n_k^U}{1 + h \sum_k \theta_{ik}^{HU} n_k^U} \right). \quad (5)$$

where the handling time $h$, of the Holling-Type II mutualistic functional response is set to $0.1$. Simulations were performed by integrating the system of ordinary differential equations using a fourth-order Runge-Kutta method.

## Optimization process

We consider a rewiring adaptation process that follows the approaches in [22, 9]. At constant time intervals, species will rewire recurrently in order to maximize their individual abundances.

1. Rewiring: At each time step $t = mT$ ($m$ is a positive integer and $T$ is the integration time), a random species $u$, with a least one link, is selected and rewired to a randomly selected species $h'$, removing one of its previous links $h$, with probability $p_{uh} \propto 1 - k_h^{-1}$. The rewiring probability is defined in such a way that the larger the species' degree is, the more prone to losing links. Once the rewiring is completed, we recalculate the mutualistic interaction factor of the new pair of nodes $\gamma_{ij'} = \Omega_m \cdot \theta_{ij'} G_{ij'}$ and integrate the dynamics according to Eq. 5, until the abundances of all species reach an equilibrium (integration time $T$ is set sufficiently large).

2. Link recovery: At the end of each time step $t$, we compare the actual abundance of species $u$ with its previous value. If the current abundance is greater than the previous value, the current (new) link is kept; otherwise, the previous one is recovered. Note that, in the case of abundance loss, only the connections are rolled back to the situation in $t - 1$; however, the vectors of abundances continue from their current state, $\vec{n}^U(t)$ and $\vec{n}^H(t)$.

## Introduction of external events

Finally, we wanted to explore how the system responds to the introduction of external events that temporarily shifts the population's attention. We modelled this situation as the change of every user's niche center towards a single common topic for a limited period of time. After that period of time, users slowly were moved back to their original niche centers, i.e. back to their respective topics.

An event modifies each users' niche in the following way:

$$G_i^E(s) = [1 - f(t_E)]G_i(s) + f(t_E)G^{E'}(s), \quad (6)$$

That is, a user's niche is now the composition of two Gaussian niches: one corresponding to the general event $E$ (defined as a new niche profile $G^{E'}(s)$ centered at $\overline{s}_E$ and width $\sigma_E$), and the original one corresponding to the user's intrinsic interests $G_i(s)$. In this formulation, $f(t_E)$ is the function that governs the growth and decay of the external event, depending on the time $t_E$ since its onset. In this work, we modelled two profiles, see Supplementary Fig. 8, along the lines of Lehmann *et al.* [14].

1. **Sudden event:** The first event considered for study was modelled as a sudden and unexpected one. In this case $f(t_E)$ take the form

$$f(t_E) = e^{-\alpha t_E} \quad (7)$$

where $\alpha$ is the decay constant. Note that, at the onset of the event ($t_E = 0$), all users are focused on the same topic, and their niche overlap will be maximum. For sufficiently large $t_E$, namely $t_E \gg \alpha^{-1}$, the influence of the event becomes negligible.

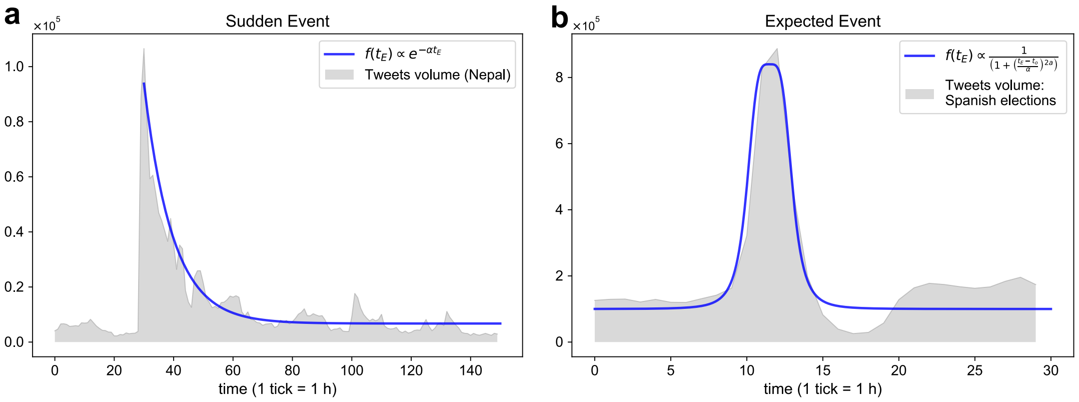


Supplementary Figure 8: **Representation of the two different type of events included in our model**. **a** shows the results a sudden and unexpected event, while **b** correspond to an expected event. To ease comparison with real scenarios, in both cases, $f(t_E)$ was shifted in order to align with the maximum and baseline activity of two empirical datasets (shadowed gray areas).

2. **Expected event:** In second place, we considered an expected event. In this case, the attention of users will slowly moves towards the one of the event, that is expected to happen an a specific time, in which the user's attention will be maximal. Here, $f(t_E)$ has the form

$$f(t_E) = \frac{1}{\left(1 + \left(\frac{t_E - t_o}{\alpha}\right)^{2a}\right)}, \tag{8}$$

where $a$ and $\alpha$ are the parameters that regulate the width of the function and the duration of the plateu, while $t_o$ specifies the location of the function peak, note that at $t_E = t_o$ the users niche overlap will be maximum. Again, we modelled the event such that for sufficiently large $t_E$, the influence of the event becomes negligible.

## Structural measures over the synthetic matrices

In same manner as in the empirical case, for the synthetic networks we will explore their structural evolution by means of nestedness [18, 4], modularity [16, 5] , and in-block nestedness [15] architectural patterns. As stated above, the explicit definitions of the three measures, employed in our analysis, are available in the main text.

## Results over the model's local parameters

To generalize the results presented in the main text, and to explore if the observed structural transitions are sensitive to changes in the model's local parameters, we perform controlled numerical experiments at the stable stationary state on the $(\Omega_m, \Omega_c)$ parameter space, for different values of the inter-intra competition parameter $\lambda$. We set both $\Omega_m$ and $\Omega_c$ in the interval $[0.1, 0.4]$ and perform simulations for 1200 different combinations of these parameters, for each value of $\lambda$.

To avoid excessive computational costs, we consider small synthetic networks of $N_U = 100$ users and $N_H = 100$ hashtags with random connections across guilds, and density (connectance) $C_o \sim 10^{-2}$. We do so to match the same order of magnitude of empirical networks when we take $N_U = 100$, see blue triangles in Supplementary Fig. 9. We assign the same initial abundance $n_0 = 0.2$ to all the users and hashtags, and the same intrinsic growth rates $\rho_U = \rho_H = 1$. Results in the following subsections correspond to an average over 10 different realisations for each combination of these parameters.

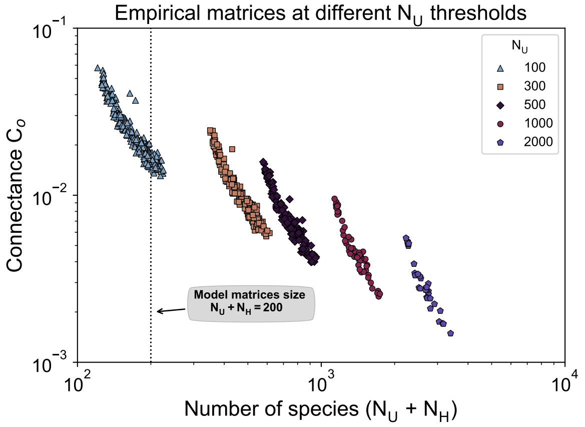


Supplementary Figure 9: Connectivity as a function of the number of species $(N_U + N_H)$ for the empirical networks, at different $N_U$ thresholds. Note the log-log scale.

1. **Pre-event steady state:** In first place, we pay attention to the abundances of individual species along the simulation time, and check for regions on the parameter space where extinctions may occur. We consider that a species goes extinct if its abundance falls below $10^{-4}$.

   Supplementary Fig. 10 shows the fraction of especies survival in the two dimensional plot in the $\Omega_m - \Omega_c$ parameter space. For all the cases, we observe that as $\Omega_m$ and $\Omega_c$ increase, extinctions start to occur, even for favorable configurations of the system in which $\Omega_m > \Omega_c$.

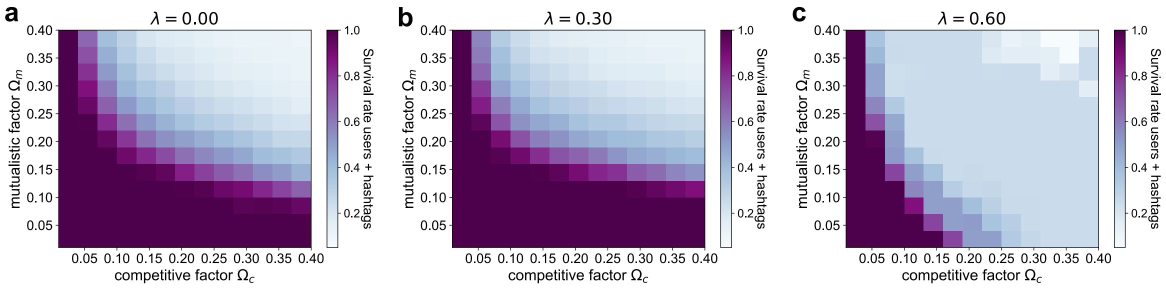


Supplementary Figure 10: **Survival rate at the pre-event steady state:** two-dimensional plots in the $\Omega_m - \Omega_c$ parameter space showing steady survival rate of the species for different values of the inter-intra competition parameter: $\lambda = 0$ (panel **a**), $\lambda = 0.3$ (panel **b**), and $\lambda = 0.6$ (panel **c**).

As expected, for low values of the inter-intra competition parameter $\lambda = 0$ and $\lambda = 0.3$ the region in which extinction do not occur is wider. Under this configuration, the species compete more strongly within their topics, which correspond to just a fraction of all the species on the system. On the contrary, as we increase $\lambda$, the region of extinctions may increase, since now each specie start to compete with a higher fraction of the system, making the system more susceptible to the values $\Omega_m, \Omega_c$. In order to guarentee the maximal survival species in the system prior to the introduction of the events, from now on, we will restrict our exploration on the $\Omega_m - \Omega_c$ to the interval $[0.01, 0.1]$. For the sake of simplicity, we will show the results just for $\lambda = 0.6$, which corresponds to the case presented in the main text.

Supplementary Fig. 11 presents the two dimensional plot in the $\Omega_m - \Omega_c$ parameter space before the event, each point within the plot corresponds to the values for each structural measure minus the values of $\mathcal{N}_o$ and

$Q_o$ measured at the beginning of the simulation. We found that the system becomes highly modular for a wide range of the $\Omega_m - \Omega_c$ values, while nestedness remain low, except for the small regions where species extinctions occur. These results are robust for all the values of $\lambda$ that were considered. Hence, by introducing species niche aligned to a certain number of topics, we were able to mimic the prescribed organisation in topical blocks observed in the empirical datasets. The modular architecture arises from the random one at the end of the optimization process.

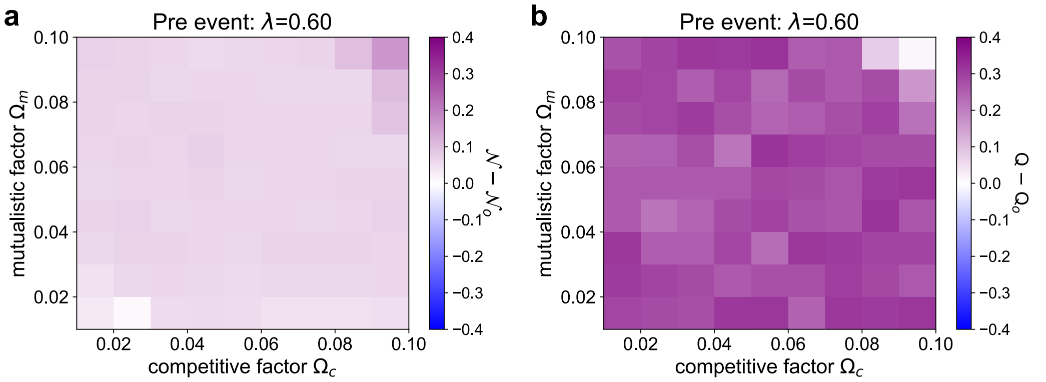


Supplementary Figure 11: **Structural measures at the pre-event steady state:** two-dimensional plots in the $\Omega_m - \Omega_c$ parameter space showing the evolution $\mathcal{N}$ (panel **a**) and Q (panel **b**) before the external event for $\lambda = 0.6$. $\mathcal{N}_o$ and $Q_o$, correspond to the values at the beginning of the simulation.

2. **Introducion of an external event:** Here, we present the results of the simulation after the introduction of an external event that correspond to a shift in the users' niches. Since we observe an equivalent structural behavior after introducing different types of events, the following plots only show the results of the case of the sudden event described in section 1.

   As shown in Supplementary Fig. 12a, for $\lambda = 0$ a considerable number of especies goes extinct by the end of the simulation time. This result is not surprising, since at the onset of the event the single topic configuration increases the competition among species. The $\lambda$ parameter helps to balance the intense competition between the species, therefore, we observe a decrease on the amount of extinctions as $\lambda$ goes higher.

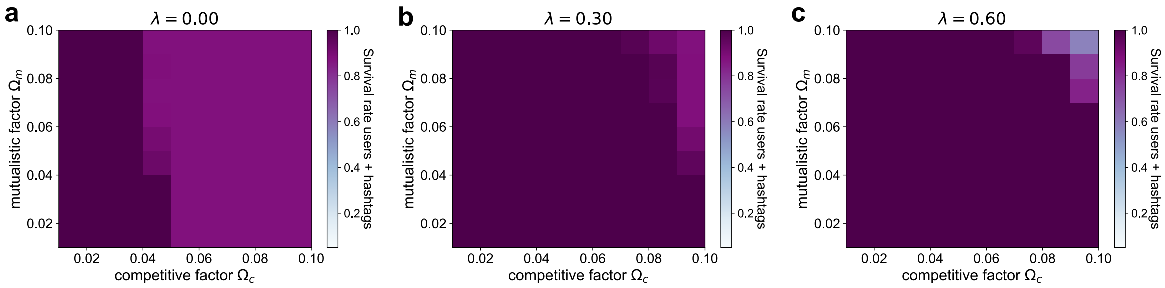


Supplementary Figure 12: **Survival rate at $t > t_E$:** two-dimensional plots in the $\Omega_m - \Omega_c$ parameter space showing survival rate of the species at the end of the simulation, for different values of the inter-intra competition parameter: $\lambda = 0$ (panel **a**), $\lambda = 0.3$ (panel **b**), and $\lambda = 0.6$ (panel **c**).

Turning our attention to the structural evolution of the system, we observe that this single topic configuration induced by the introduction of the event, provokes a structural transition from modular to a global nested

pattern on the system, for a wide range of the $\Omega_m - \Omega_c$ parameters, see Supplementary Fig 13. Again, in Supplementary Fig 13, $\mathcal{N}_o$ and $Q_o$ correspond to the values for nestedness and modularity at the eginning of the simulation. We observe that modularity drops abruptly, while nestedness increases. This result is in accordance with our empirical observations reported above and in the maint text.

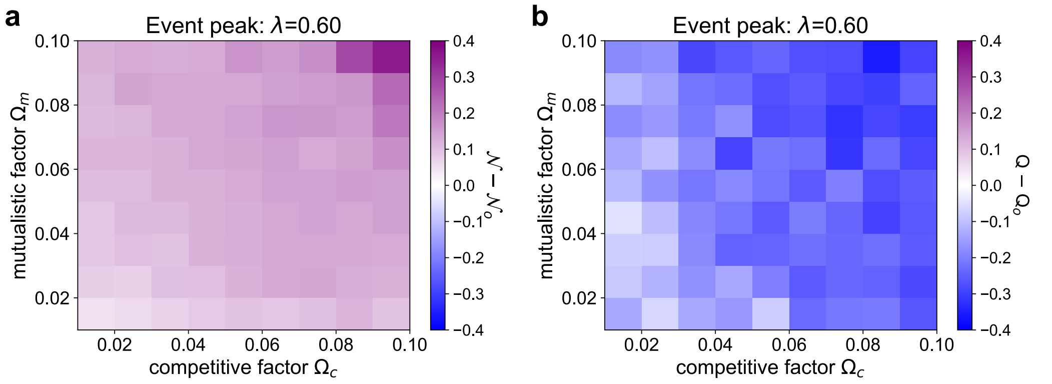


Supplementary Figure 13: **Structural measures at** $t > t_E$**:** Two-dimensional plots in the $\Omega_m - \Omega_c$ parameter space showing the evolution of $\mathcal{N}$ (panel **a**) and Q (panel **b**) after the external event ($\lambda = 0.6$). $\mathcal{N}_o$ and $Q_o$, correspond to the values at the beginning of the simulation.

3. **From meso- to macroscale nested arrangements:** Finally, we also explored the structural evolution of the system by means of the in-block nestedness function $\mathcal{I}$ [20]. This exploration confirms the general character of the fluctuating nested self-similar organization. Supplementary Figure 14 shows the relative size of the largest nested blocks $N_{B_1}(\mathcal{I})/N$, before (panel (a)) and after (panel (b)) the external event. Before the event, we observe that in general, for all the parameter space, the size of the largest nested block constitutes a $25\%$ of the whole network, approximately, i.e, the user are evenly aligned over the four predefined topics. After the event, we observed how a state of global consensus is emerging, as $N_{B_1}/N$ increases over all the parameter space, representing more than $50\%$ of the size of the network in most of the cases.

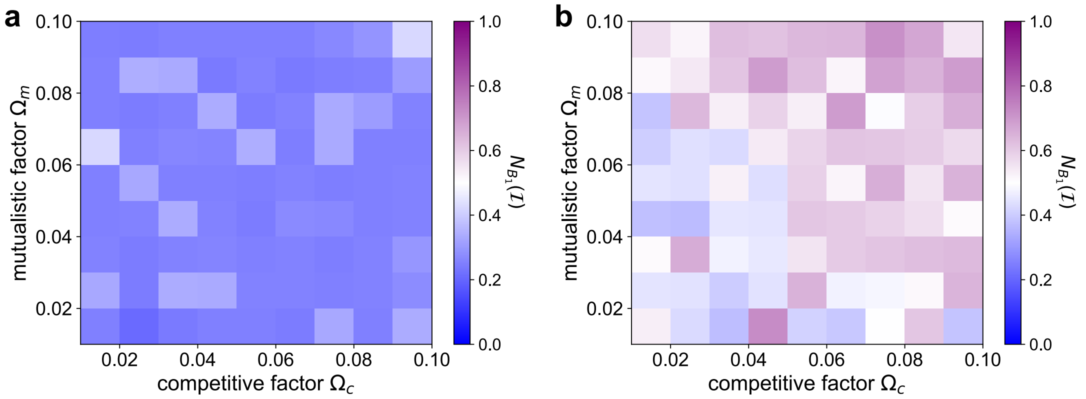


Supplementary Figure 14: **Relative size of the largest nested block** $(N_{B_1}/N)$ **before and after the external event:** two-dimensional plots in the $\Omega_m - \Omega_c$ parameter space showing the relative size of the largest nested block before (panel **a**) and after (panel **b**) the external event with $\lambda = 0.6$.

## Supplementary Note 3: Model-data agreement: further comparisons

In the main text and previous sections, we have shown that the proposed model is able to reproduce the structural macro- and mesoscale fluctuations observed in the empirical data, as well as some microscopic features. In this Supplementary Note, we strengthen the connection between the model and the data, from a broader quantitative and qualitative point of view.

### Anti-correlated behaviour between $Q$ and $\mathcal{N}$

In this section, we discuss in depth the observed anti-correlated behaviour between nestedness and modularity, for both the empirical data and the model's outcome.

We know from previous studies, that there exists an upper bound for the co-existence of nested and modular structures, regardless of the size or the density of the network at stake [17]. This bound is $Q \leq 1 - \mathcal{N}$, and implies that a highly modular structure can only "afford" a non-nested structure, and the other way around, which helps to explain the observed anti-correlated behaviour between $Q$ and $\mathcal{N}$. Here, we statistically confirm such anti-correlated behaviour between $Q$ and $\mathcal{N}$ by computing the Pearson correlation between both measures across time, see Table 2. For the synthetic cases (last two rows), it is measured from $t = 1.5 \times 10^4$ onwards, to avoid the initial random fluctuations. For smoother comparisons, correlation is measured over the whole period covered for the datasets, but also before/during/after the main events that we identify in Fig. 1 of the main text, and Supplementary Fig. 4, respectively. Except for the Catalan dataset, the matching between empirical and synthetic results is remarkable, not only in the periods where the correlation is strong, but also during the pre-event stages, where, in most cases, both correlations are irrelevant, despite their opposed signs.

The mismatch in the Catalan dataset, can also be explained in terms of the upper bound described above, since such bound does not rule out other possible regimes. For example, it is quite common for both $Q$ and $\mathcal{N}$ values in a network to be extremely low; or that both have intermediate values, which typically signals the presence of in-block nestedness. The Catalan political conflict has an associated extremely high polarization: the dataset contains both users in favour of and against a referendum, and a large fraction of Spanish users who think that a referendum should not even be discussed at all. As a consequence, the polling day, for example, contains not only tweets paying attention to the results of the (illegalised) referendum, but also many messages calling for political and legal action against the organizers, or simply appealing to political dialogue between the parts. In structural terms, all of this translates into an in-block nested structure, suggesting a sort of "partial consensus" among the different sides that participate in the conversation. Therefore, for this case, we can obtain intermediate values of both quantities ( $Q$ and $\mathcal{N}$), which explains the weak anti-correlation observed that day ($r = -0.3079$).

### Statistical significance of $Q$ and $\mathcal{N}$

To further strengthen the validity of our results, we now are focused on exploring a possible lack of statistical significance of the reported patterns $Q$ and $\mathcal{N}$, which has been and is a controversial issue for these descriptors. Before explaining the randomisation procedure employed to assess the statistical significance of $Q$ and $\mathcal{N}$, we want to stress here that, by definition, both descriptors incorporate a null model term. While this is not new for $Q$, whose quantification has always been in reference to a null term, it is so for the definition of $\mathcal{N}$ that we employ, which differs from the classical ones (e.g. NODF [3]), that do not include a random expectation term; see eq. (1) in the main text.

For the sake of simplicity, and due to computational limitations, we only perform the statistical test –and all the further comparisons– for the Spanish dataset. For each one of the matrices of the Spanish dataset and the synthetic matrices under the expected event, we have generated 150 randomizations in which we preserve the link density. This form of randomisation amounts to considering that the overall system's activity is kept, but users lack a preference for one or another meme for communication purposes. Supplementary Figure 15 shows the results of the $z$-scores of the two descriptors against the ensemble of randomized matrices. The black solid lines in the plots

Supplementary Table 2: Pearson coefficients for $Q$ and $\mathcal{N}$ at different times, for both, the model and the data.

| Data type | Dataset | Whole period | Pre event | Event | Post event |
|---|---|---|---|---|---|
| **Empirical** | 2019 Spanish general elections | -0.8264 | -0.2914 | Debate: -0.9094<br>Polling: -0.7178 | -0.8467 |
| | 2015 Nepal Earthquake | -0.7337 | -0.26566 | -0.9358 | -0.74138 |
| | 2014 Catalan referendum | -0.1490 | -0.1036 | Diada: -0.21326<br>Polling: -0.3079 | -0.7234 |
| | 2012 UEFA football championship | -0.7930 | -0.42895 | Semis: -0.8675<br>Finals : -0.7545 | -0.8827 |
| | 2014 Hong Kong Protests | -0.5774 | 0.0034 | Occupy central: -0.6194 | -0.6180 |
| | 2015 Charlie Hebdo shooting | -0.9180 | -0.4496 | -0.8979 | -0.8240 |
| **Numerical** | "expected event" | -0.743 | 0.1625 | -0.7611 | -0.8641 |
| | "sudden event" | -0.6651 | -0.0558 | -0.7390 | -0.8533 |

corresponds to a $z = 2$. The dotted lines show the actual $Q$ and $\mathcal{N}$ values on the real matrices, that are indicated by the secondary $y-$axis in both panels.

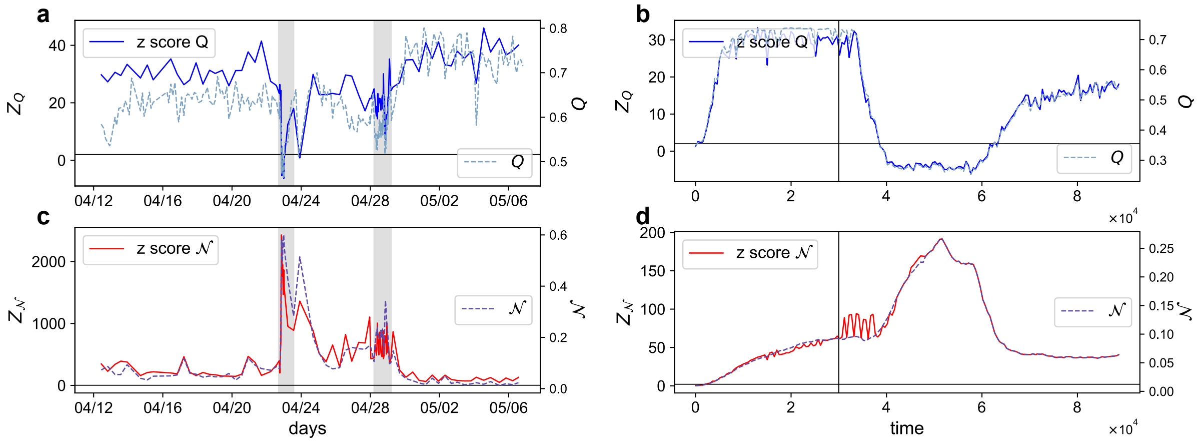


Supplementary Figure 15: **z-scores for modularity and nestedness, against an ensemble of 150 randomisations:** for the empirical case (panel **a** and **c**) and for the model's numerical simulation (panel **b** and **d**).

From Supplementary Fig. 15a, we observe that during the debate the measured values for $Q$ are no longer statistically significant at the selected confidence interval. For the second event (polling), we observe an abrupt decrease in the $z$-scores for the measured $Q$, although it is still significant under the considered threshold. In the case of $\mathcal{N}$ (Supplementary Fig. 15c), we observe that its values remain statistically significant for the whole dataset. Nonetheless, we find evident variances between their statistical significance during the crucial periods. In general, the $z$-scores for $\mathcal{N}$ are extremely high during the extreme event, despite low values (compared to the peak) outside the exogenous events. Pursuing stronger connections between findings in the data and the model, we have also analyzed, under the same scope, the outcome of our model. Results over the synthetic networks (Supplementary Fig. 15b and d), show the same observed behaviour: during the artificially introduced event, the $z$-scores for $Q$ fall below statistical significance; while $\mathcal{N}$ is significant overall the period, but with a marked surge

after the exogenous introduction of an event at $t = 3 \times 10^4$, proving that, in general, the changes in $Q$ and $\mathcal{N}$ are beyond reasonable expectation, and are thus statistically robust.

This last statement is further confirmed by applying a second approach to assess the significance of the structural transitions. Specifically, we have verified such significance by comparing the results with respect to the median in the time series, see Supplementary Fig. 16. For the Spanish dataset and the numerical simulations, we have measured the median and interquartile range (IQR), solid line and shaded areas, respectively. For the empirical case Supplementary Fig. 16 (a), the $Q$ values fall below $Q-$IQR during the two large events considered (debate and election day), implying a large deviation from its baseline. With respect to the $\mathcal{N}$ (Supplementary Fig. 15c), we observe that their values remain below the reasonable expectation most of the time, except during the debate episode and during election day, although in the latest the increase is moderate. The same can be said considering the results of our model: $Q$ only falls below expectation after the artificial introduction of an event (at $t = 3\times10^4$), and lasts approximately $4 \times 10^4 < t < 6 \times 10^4$, while $\mathcal{N}$ increases within the range $4 \times 10^4 < t < 6 \times 10^4$.

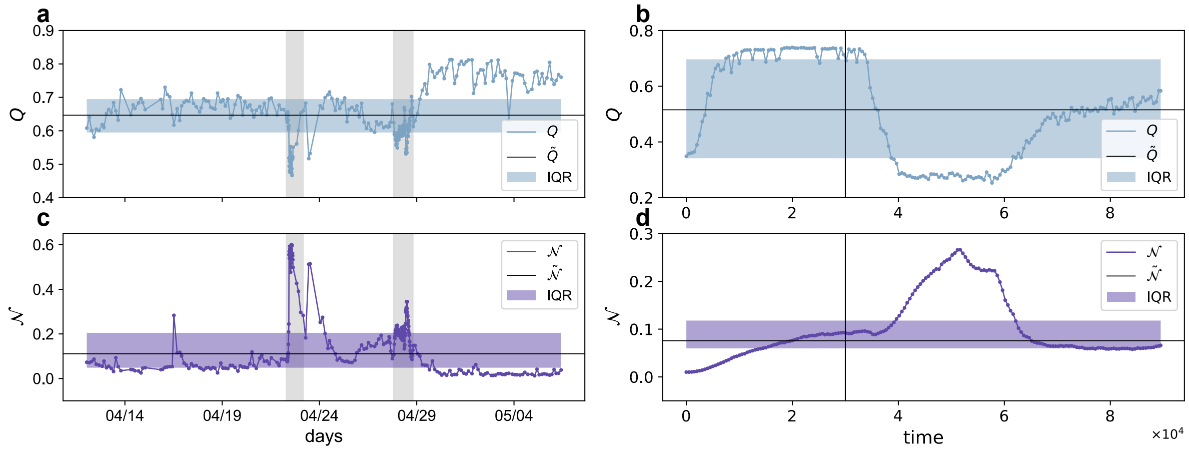


Supplementary Figure 16: **Median and interquartile range (IQR) of the structural measures:** for the Spanish dataset (panels **a** and **c**) and the model's outcome (panel **c** and **d**). The median is shown as a solid horizontal line, and the shaded area represents the IQR. For both cases, $Q$ (**a** and **b**) falls below expectation during the exogenous events. On the opposite, $\mathcal{N}$ increases for those times in which exceptional events occur (**c** and **d**).

## Disentangling the effects of a change in the activity

In this section, we continue deepening the connections between the model and data, by exploring in detail the effects of the activity changes on the system's properties. Our main interest in exploring this particular aspect is to avoid a possible confounding factor: the false impression that changes in activity can, on their own, explain the reported structural shifts from (to) modular to (from) nested arrangements. In other words, we intend to discard the idea that changes in the network's topology are simple by-products of changes in the activity.

Examining this aspect in more detail, we can observe that most of the increased activity during extraordinary events is due to new users entering the topic (Supplementary Fig. 17a), and that these produce a very large amount of hashtags as well (Supplementary Fig. 17b). Results from Supplementary Fig. 17 may be interpreted as a proof that the model cannot mimic the observed behaviour in the data, e.g. the number of user and hashtags is highly fluctuating in the data, while it remains constant in the model. In the following section, however, we explore whether other system's quantities, such like the users' average activity or the amount of effective hashtags, are also affected by activity increases.

1. **Effects of activity increase on the users' and hashtags average quantities:** We start by exploring the effect of an activity increase from the users' perspective. Specifically, we tracked the users' average activity,

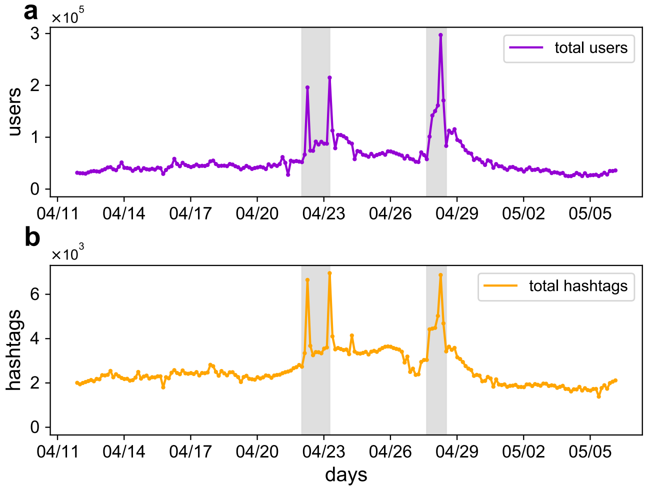


Supplementary Figure 17: Evolution of the number of users **a** and the the number of hashtags during the Spanish election cycle °**b**. Both quantities show remarkable increases during the identified exceptional events (debate, polling day).

i.e., the number of hashtags per user, $\langle h\rangle$, over time (Supplementary Fig. 18a). We can observe that, even during an exceptional event, $\langle h\rangle$ remains relatively constant, this means that when the users' attention profiles are shifted, these do not significantly increase their activity (in terms of hashtag usage) on average, but rather start switching towards the topic on which that same activity is devoted. This finding provides a stronger link between actual data and the model for which $\langle h\rangle$ is constant by design, as can be seen from Supplementary Fig. 18b.

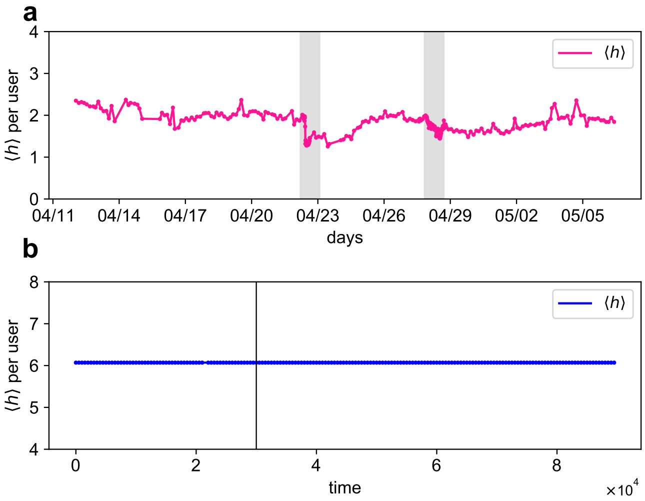


Supplementary Figure 18: **Average number of hashtags per user** $\langle h\rangle$**:** for the Spanish dataset (panel **a**), and for the model's numerical simulation (panel **b**).

Moving on, we have also explored the effects of an activity increase from the hashtags' side. Notably, from Supplementary Fig. 17b, it is clear that the absolute number of unique hashtags increases during the

highlighted events, debate, and polling day, respectively. Nonetheless, when we try to quantify the minimum amount of hashtags needed to account for a large fraction of the users (99% of the users, in our case) in each time window, i.e. the diversity of the hashtags in terms of a cover set, we see that 99% of the users can be accounted for with no more than 400 hashtags, and with 100 hashtags or less during intense attention episodes (Supplementary Fig. 19a). This behavior is qualitatively mimicked as well by the model, see Supplementary Fig. 19b. We have computed this "hashtag coverset" by applying the following iterative scheme: we count (and remove) all users who tweeted the most frequent hashtag; then we count (and remove) all users who used the second most frequent hashtag; and so on, until we reach the desired threshold (99% of the users). Counted in this way, the hashtag coverset represents to what extent users are focused on only a few items, despite the presence of many more memes in the information system that may (or probably may not) get anyone's attention. Last but not least, these results show that, even though the model does not take into account the fluctuating behaviour of users and hashtags observed in the data, which cannot be incorporated without considering birth/invasion processes, it is still able to reproduce the evolution of *effective* hashtags in the system.

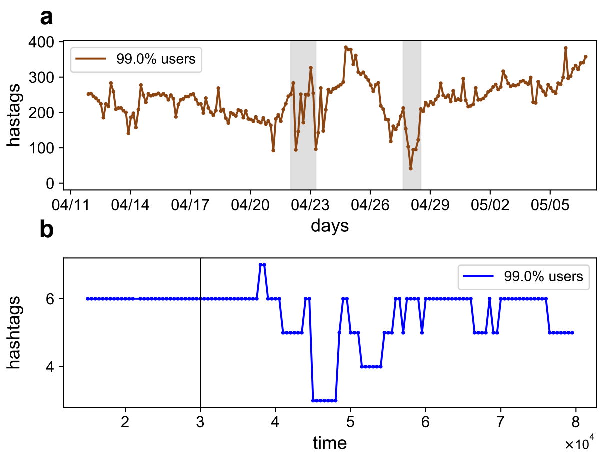


Supplementary Figure 19: **Hashtag coverset at the 99% threshold :** for the Spanish dataset (panel **a**), and for the model's numerical simulation (panel **b**).

2. **Activity increase as a driver for nestedness and/or modularity:** We now want to investigate if an increase in activity can be a driving mechanism of the different structural transitions observed in the data. As explained in Section , given the mutual constraints that $Q$ and $\mathcal{N}$ impose on each other, the growth of one implies the decline of the other. Nonetheless, the opposite is not necessarily true: a reduction in $Q$ (due, for example, to larger density in the network) does not guarantee at all an automatic increase of nestedness. Conversely, a reduction in $\mathcal{N}$ will not imply, necessarily, a larger $Q$. Since we know *a priori* that an increase in activity may lead to a growth in network density, here, we want to fully address if such increment could be responsible for the increase in nestedness/decrease in modularity observed in the model and data.

   We start our examination providing a simple example from the Spanish dataset: we take a snapshot of the system right before a large event occurs (debate), in which the system is clearly organized as a modular network and have a connectance (density) $C = 0.003$. Then, we start simulating an increase in activity (which translates to an increase in density), taking the system from the initial connectance to a final one of $0.005$. We choose this network density, as this is precisely the connectance in the empirical data by the time we observe a maximum in nestedness (during the debate).

   Supplementary Figure 20 summarises the results from the experiment. In the left panel, we see the network at its "starting point": $Q$ takes a high value $(0.677)$ while nestedness is negligible $(0.058)$. Adding activity

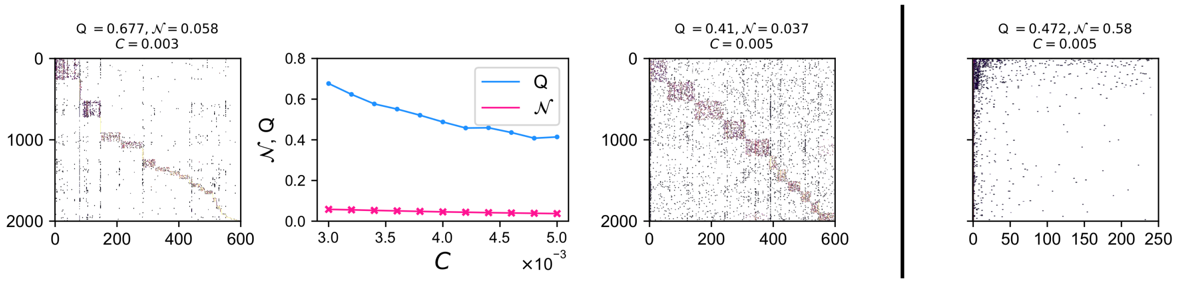


Supplementary Figure 20: **Expected effects of random increased activity in empirical data:** the system, organized in a modular pattern initially (left) transitions to a highly nested one (right); however, randomly increasing connectance (activity) does not imply per se increases in the values of nestedness (middle panels).

at random, up to $C = 0.005$, does not lead to a nested architecture. On the contrary, the process of adding links to the network has deteriorated both $Q$ and $\mathcal{N}$. The second panel of Supplementary Fig. 20 shows the evolution of both quantities as links are added, and the third panel shows the resulting network. For the sake of comparison, the panel to the right shows the actual (empirical) network at the end of the process, in which nestedness peaks at $0.58$ –an order of magnitude above what one would observe if activity is increased at random. We can observe that increased activity does not render, per se, any gains in $\mathcal{N}$ –rather, nestedness stays negligible to a value of in the order of $10^{-2}$. To better grasp this relevant point, we further extend our experiment from Supplementary Fig. 20 to more general scenarios, employing a synthetic benchmark.

Starting from an initially modular, an initially nested, and an initially in-block nested network, each one of size $N_{col} = N_{row} = 150$ nodes, we randomly increase their densities, as a null model of activity growth, i.e., users are linking to more and more hashtags at random. Supplementary Figure 21 below shows the results from this study. Each point of the plot corresponds to an increase of $5\%$ in the amount of links. From the initially modular network (top row), we observe that, as we move along the $x$-axis (added links), increasing activity at random decreases the modularity, and yet nestedness remains in the extremely low values that it showed initially. For the initially nested (middle row), and initially in-block nested (bottom row) networks, the results are very similar: no growth of the complementary pattern is observed at all.

Ultimately, it is clear that a sole increase in activity (in any situation) is not necessarily related to an implicit increment of any of the measures used in the paper. In fact, nestedness for example, may emerge without a remarkable increase in activity: see panels (a), (c) and (d) of Supplementary Fig. 4. Shifts to nestedness in these cases are not directly related to increase in activity. Although we are aware that this result clashes with the idea that connectance underlies the emergence of nestedness [13], we want to stress that such increases in nestedness related to increases in connectance are usually quantified through the use of descriptors that does not discount the amount of overlap that two species may have due to random fluctuations, e.g., Almeida-Neto's NODF [3]. Instead, in this work, we have employed a measure of nestedness, which already includes a random expectation term. All in all, our results seem to indicate that, for all the three measures, it is required that some explicit driver on the network constituents and their interactions guides the changes at the macroscopic or mesoscopic levels.

## Data-model agreement over basic network statistics

Furthermore, it is important that we perform data-model comparisons over basic network statistics, in order to further validate the robustness of our theoretical framework.

Already in Supplementary Fig. 18, we showed a comparison between the users' average degree $\langle h \rangle$ for the synthetic networks and the Spanish dataset. As a reminder, while the $\langle h \rangle$ in the model is constant by design, it is in

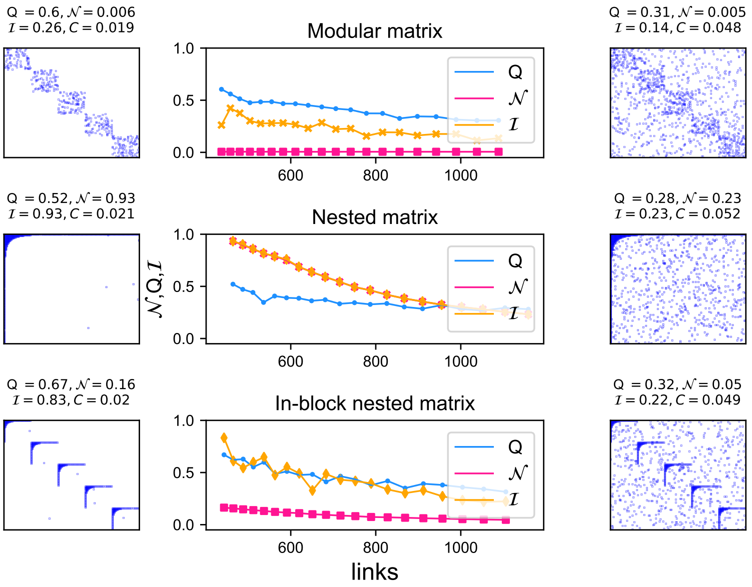


Supplementary Figure 21: **Effects of randomly increasing density on synthetic networks** with purely modular (top), nested (middle) and in-block nested (bottom) organizations. Random link addition harms the idealised initial structures, while it has no positive effects on the other descriptors.

excellent agreement with the empirical case, where the $\langle h \rangle$ remains relatively constant, even during the exceptional events. Going forward, in this section, we introduce as well a comparison between the observed (Spanish dataset) and the model's degree distributions, see Supplementary Supplementary Figure 22. Notably, the degree distribution in different states of the system for the synthetic case is also in good agreement with the empirical results.

Finally, we want to remark that further quantitative comparisons are difficult to perform, since our synthetic approach represents an idealization of actual systems: not only our toy model has an arbitrarily small size (although it preserves the scaling of connectance, see Supplementary Supplementary Fig. 9), but also we impose a small amount of topics, which, on top of that, are equally sized (exactly a fourth of the synthetic users and memes are centered around each topic). Clearly all of this represents an idealisation of actual systems, and therefore some specific empirical particularities cannot be matched.

## Species turnover in the model

Supplementary Note 1 and Supplementary Figure 2 above quantify the temporal continuity of users and hashtags over time. At the face of it, a question can arise about the failure of the model to include this feature in particular, that is, the fact that empirical systems experience a high species turnover. Here, we provide some evidence against such question.

The first fact that we need to take into account is that, in our synthetic simulations, we always have a privileged global view of the system, in which nodes cannot enter or leave the observed stage. That is, $Q$, $\mathcal{N}$ and $\mathcal{I}$ are measured including all the nodes and their interactions. For a fair comparison with the data in this aspect, we should consider a fraction of the synthetic system, and then see whether the top users (or hashtags) do change it time or not.

This is what Supplementary Supplementary Figure 23 precisely shows. To obtain it, we take the whole history of the synthetic simulation shown in Figure 3 of the main text. Keep in mind that the system is made up of $N_U = N_H = 100$. Here, as we do with empirical data, we assume that we can only manage a partial observation

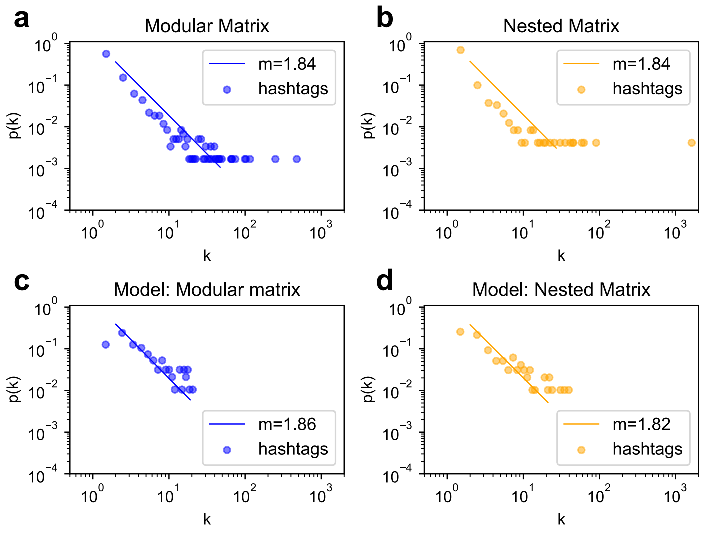


Supplementary Figure 22: **Comparative analysis of the hashtags degree distributions.** Panels **a** and **b** correspond to the results for the Spanish dataset during the pre-event stage (left column), which presents a modular organization, and during the event stage (debate), which presents a nested organization, respectively. Panels **c** and **d** present the equivalent results for the numerical simulations.

of the system, so for each temporal slice we select the 50 most active (abundant) users, and the corresponding hashtags that they cite (which may be any number from 0 to 100). In the figure, we show the overlap between slices for hashtags (Supplementary Fig. 23a) and users (b). We can see that these overlaps are, in general, smaller than 1; and these changes are even more drastic after the introduction of an external shock ($t = 3 \times 10^4$), when the overlaps hit the minimum.

The fact that the overlap in this experiment is significantly larger than the empirical one, i.e. Supplementary Figure 2, is an effect of the small size of the synthetic experiment (both in terms of nodes, and number of topics). We remark that here we are systematically considering half of the users (and often more than half of the hashtags), which is not the case in general when analysing empirical datasets (see Supplementary Figure 24 below).

Finally, we highlight that the partial pruning of the system does not affect the observation of emergent structural patterns: Supplementary Fig. 24 displays the evolution of $Q$ and $\mathcal{N}$, which closely follow the trends in Figure 3 of the main text.

Summarising, the apparent failure of the model to match the observed species turnover is a consequence of the limited computational capabilities: synthetic simulations, unlike real datasets, always permit a privileged global view of the system, in which nodes cannot enter or leave the observed stage. That said, we insist that the model needs, in future efforts, to include birth/death/invasion processes, because species turnover in empirical data is not just a matter of network pruning (i.e. which nodes are included in the network), but also of nodes that join and leave the system (if only because of circadian rhythms).

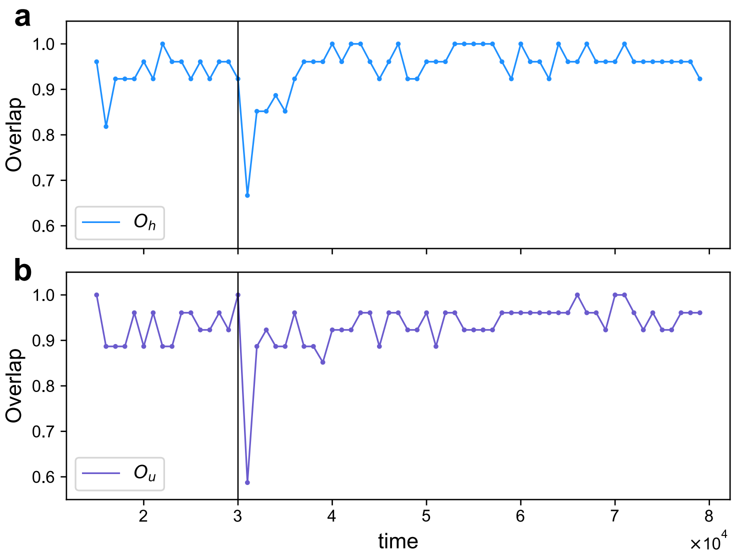


Supplementary Figure 23: **Partial view of the synthetic system (I).** Overlap across time between the hashtags (panel **a**) and users (panel **b**) in consecutive snapshots, when only the top 50% of the users are taken into account.

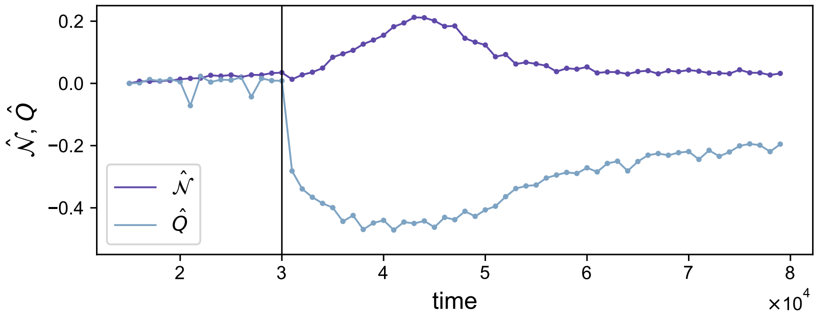


Supplementary Figure 24: **Partial view of the synthetic system (II).** The partial view of the system does not blur the emergent structural patterns ($Q$ and $\mathcal{N}$) reported in Figure 3 of the main text.

# Supplementary Note 4: Optimization algorithms for $\mathcal{I}$: sensitivity test

Contrary to the case of modularity, where a rich collection of algorithms for its optimization are currently available, dedicated methods to optimize the in-block nestedness function, which was just recently introduced [20], are still very scarce. From a computational point of view, searching for a (sub)optimal in-block nested partition is very similar to searching for a modular one: the choice of a maximization heuristic tries to balance computational speed with accuracy. In this work, we have employed the extremal optimization algorithm [12] to perform the maximization modularity and in-block nestedness, we chose this algorithm since it is known to provide a very convenient balance between accuracy and cost [10].

To prove this point, in this Supplementary Note, we compare our optimization results to an alternative heuristics, the bee swarm optimization algorithm, [20], which is known to deliver high-accuracy results at the cost of a large computational effort. Precisely because of this high cost, we cannot present a comparison based on the Twitter networks of the manuscript, but rather on a collection of smaller networks. Particularly, we have compared the two algorithms for a collection of 140 ecological networks, available at [1]. Supplementary Figure 25, shows a scatter plot of the obtained values of $\mathcal{I}$ after each optimization process. The solid blue line corresponds to the linear fit, and the solid orange line corresponds to $y = x$. We computed the Pearson correlation coefficient (Pearson= $0.806$) and highlighted in red the networks for which the difference between the two values is less than $0.05$. In general, we observed a good agreement, and a high correlation between the $\mathcal{I}$ values obtained from each strategy. As expected, since the bee swarm algorithm is more exhaustive, it usually delivers higher $\mathcal{I}$ values. Nonetheless, we can observe that the bee swarm heuristic performs better than EO in most small networks, but worse for the two largest networks compared (in the order of hundreds of nodes).

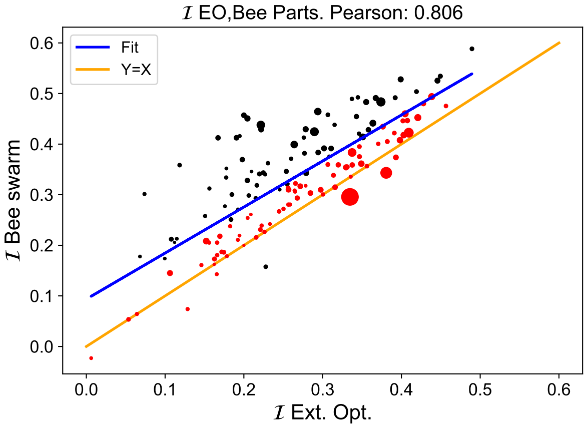


Supplementary Figure 25: **Scatter plot comparing the $\mathcal{I}$ values for two optimization strategies.** The size of the dots is propotional to the networks size. Solid blue line corresponds to the linear fit, and the solid orange line corresponds to $y = x$. Dots highlighted in red correspond to networks for which the difference between the two $\mathcal{I}$ values is less than $0.05$.

## Supplementary References


[1] Web of Life: ecological networks database. `http://www.web-of-life.es/`, 2012.

[2] Ruben Alarcón, Nickolas M Waser, and Jeff Ollerton. Year-to-year variation in the topology of a plant–pollinator interaction network. *Oikos*, 117(12):1796–1807, 2008.

[3] Mário Almeida-Neto, Paulo Guimaraes, Paulo R Guimarães, Rafael D Loyola, and Werner Ulrich. A consistent metric for nestedness analysis in ecological systems: reconciling concept and measurement. *Oikos*, 117(8):1227–1239, 2008.

[4] Wirt Atmar and Bruce D Patterson. The measure of order and disorder in the distribution of species in fragmented habitat. *Oecologia*, 96(3):373–382, 1993.

[5] Michael J Barber. Modularity and community detection in bipartite networks. *Physical Review E*, 76(6):066102, 2007.

[6] Jordi Bascompte, Pedro Jordano, Carlos J Melián, and Jens M Olesen. The nested assembly of plant–animal mutualistic networks. *Proceedings of the National Academy of Sciences*, 100(16):9383–9387, 2003.

[7] Nico Blüthgen, Jochen Fründ, Diego P Vázquez, and Florian Menzel. What do interaction network metrics tell us about specialization and biological traits. *Ecology*, 89(12):3387–3399, 2008.

[8] Javier Borge-Holthoefer, Raquel A Baños, Carlos Gracia-Lázaro, and Yamir Moreno. Emergence of consensus as a modular-to-nested transition in communication dynamics. *Scientific Reports*, 7:41673, 2017.

[9] Weiran Cai, Jordan Snyder, Alan Hastings, and Raissa M D'Souza. Mutualistic networks emerging from adaptive niche-based interactions. *Nature communications*, 11(1):1–10, 2020.

[10] Leon Danon, Albert Diaz-Guilera, Jordi Duch, and Alex Arenas. Comparing community structure identification. *Journal of Statistical Mechanics: Theory and Experiment*, 2005(09):P09008, 2005.

[11] Cecilia Díaz-Castelazo, Paulo R Guimaraes Jr, Pedro Jordano, John N Thompson, Robert J Marquis, and Víctor Rico-Gray. Changes of a mutualistic network over time: reanalysis over a 10-year period. *Ecology*, 91(3):793–801, 2010.

[12] Jordi Duch and Alex Arenas. Community detection in complex networks using extremal optimization. *Physical Review E*, 72(2):027104, 2005.

[13] Alex James, Jonathan W Pitchford, and Michael J Plank. Disentangling nestedness from models of ecological complexity. *Nature*, 487(7406):227–230, 2012.

[14] Janette Lehmann, Bruno Gonçalves, José J Ramasco, and Ciro Cattuto. Dynamical classes of collective attention in twitter. In *Proceedings of the 21st international conference on World Wide Web*, pages 251–260. ACM, 2012.

[15] Thomas M Lewinsohn, Paulo Inácio Prado, Pedro Jordano, Jordi Bascompte, and Jens M Olesen. Structure in plant–animal interaction assemblages. *Oikos*, 113(1):174–184, 2006.

[16] Mark EJ Newman and Michelle Girvan. Finding and evaluating community structure in networks. *Physical Review E*, 69(2):026113, 2004.

[17] María Palazzi, Javier Borge-Holthoefer, Claudio Tessone, and Albert Solé-Ribalta. Macro-and mesoscale pattern interdependencies in complex networks. *Journal of the Royal Society Interface*, 16(159):20190553, 2019.

[18] Bruce D Patterson and Wirt Atmar. Nested subsets and the structure of insular mammalian faunas and archipelagos. *Biological Journal of the Linnean Society*, 28(1-2):65–82, 1986.

[19] Theodora Petanidou, Athanasios S Kallimanis, Joseph Tzanopoulos, Stefanos P Sgardelis, and John D Pantis. Long-term observation of a pollination network: fluctuation in species and interactions, relative invariance of network structure and implications for estimates of specialization. *Ecology letters*, 11(6):564–575, 2008.

[20] Albert Solé-Ribalta, Claudio J Tessone, Manuel S Mariani, and Javier Borge-Holthoefer. Revealing in-block nestedness: detection and benchmarking. *Physical Review E*, 96(6):062302, 2018.

[21] Phillip PA Staniczenko, Jason C Kopp, and Stefano Allesina. The ghost of nestedness in ecological networks. *Nature communications*, 4:1391, 2013.

[22] Samir Suweis, Filippo Simini, Jayanth R Banavar, and Amos Maritan. Emergence of structural and dynamical properties of ecological mutualistic networks. *Nature*, 500(7463):449, 2013.

[23] Konstantina Zografou, Mark T Swartz, Virginia P Tilden, Erika N McKinney, Julie A Eckenrode, and Brent J Sewall. Stable generalist species anchor a dynamic pollination network. *Ecosphere*, 11(8):e03225, 2020.

[24] Arkaitz Zubiaga. A longitudinal assessment of the persistence of twitter datasets. *Journal of the Association for Information Science and Technology*, 69(8):974–984, 2018.
