# Supplementary material for: An ecological approach to structural flexibility in online communication systems
Source: Nat Commun. 2021 Mar 29;12:1941. doi: 10.1038/s41467-021-22184-2 (PMC8007599; doi:10.1038/s41467-021-22184-2)
Supplement: Supplementary file 3 — Reporting Summary [file 41467_2021_22184_MOESM3_ESM.pdf]

nature research

Corresponding author(s): Javier Borge-Holthoefer

Last updated by author(s): Feb 22, 2021

Reporting Summary

Nature Research wishes to improve the reproducibility of the work that we publish. This form provides structure for consistency and transparency in reporting. For further information on Nature Research policies, see our Editorial Policies and the Editorial Policy Checklist.

Statistics

For all statistical analyses, confirm that the following items are present in the figure legend, table legend, main text, or Methods section.

n/a	Confirmed	
☐	☒	The exact sample size (n) for each experimental group/condition, given as a discrete number and unit of measurement
☐	☒	A statement on whether measurements were taken from distinct samples or whether the same sample was measured repeatedly
☒	☐	The statistical test(s) used AND whether they are one- or two-sided<br>*Only common tests should be described solely by name; describe more complex techniques in the Methods section.*
☒	☐	A description of all covariates tested
☒	☐	A description of any assumptions or corrections, such as tests of normality and adjustment for multiple comparisons
☐	☒	A full description of the statistical parameters including central tendency (e.g. means) or other basic estimates (e.g. regression coefficient) AND variation (e.g. standard deviation) or associated estimates of uncertainty (e.g. confidence intervals)
☒	☐	For null hypothesis testing, the test statistic (e.g. F, t, r) with confidence intervals, effect sizes, degrees of freedom and P value noted<br>*Give P values as exact values whenever suitable.*
☒	☐	For Bayesian analysis, information on the choice of priors and Markov chain Monte Carlo settings
☒	☐	For hierarchical and complex designs, identification of the appropriate level for tests and full reporting of outcomes
☐	☒	Estimates of effect sizes (e.g. Cohen's d, Pearson's r), indicating how they were calculated

Our web collection on statistics for biologists contains articles on many of the points above.

Software and code

Policy information about availability of computer code

Data collection	No data collection was performed for this article. We used available datasets, which are shared as detailed in the next section (Data).
Data analysis	The software for nestedness measurement, and modularity and in-block nestedness optimisation is custom code, and available at the GitHub repository of the research group CoSIN3: https://github.com/COSIN3-UOC/

For manuscripts utilizing custom algorithms or software that are central to the research but not yet described in published literature, software must be made available to editors and reviewers. We strongly encourage code deposition in a community repository (e.g. GitHub). See the Nature Research guidelines for submitting code & software for further information.

Data

Policy information about availability of data

All manuscripts must include a data availability statement. This statement should provide the following information, where applicable:

- Accession codes, unique identifiers, or web links for publicly available datasets
- A list of figures that have associated raw data
- A description of any restrictions on data availability

The Catalan and Spanish datasets are available at OSF with the identifier DOI: 10.17605/OSF.IO/J5QWX. The rest of the datasets employed in this study were collected by Zubiaga A. (ref. 51 in the main text) and are available at Figshare with the identifier DOI:10.6084/m9.figshare.5100460.v2.

Field-specific reporting

Please select the one below that is the best fit for your research. If you are not sure, read the appropriate sections before making your selection.

☐ Life sciences ☒ Behavioural & social sciences ☐ Ecological, evolutionary & environmental sciences

For a reference copy of the document with all sections, see nature.com/documents/nr-reporting-summary-flat.pdf

Behavioural & social sciences study design

All studies must disclose on these points even when the disclosure is negative.

Study description	The work studies the structural patterns that emerge in time-resolved online communication networks, and then proposes an ecological modelling framework that can explain some of the mechanisms underlying those systems. Results on data are quantitative, and comparison between empirical and modelling results are noth quantitative and qualitative.
Research sample	For empirical analyses, the work relies on six Twitter datasets that capture the activity of millions of users on a selection of topics (Spanish electoral period in 2019, Nepal earthquake in 2015, Catalan self-determination referendum in 2014, UEFA football championship in 2012, Hong Kong protests in 2014, Charlie Hebdo Shooting in 2015). These samples are representative of those events, inasmuch almost all related activity was captured. Also, these examples are good representatives of Twitter activity, given their topical heterogeneity.
Sampling strategy	The empirical data employed in this work was collected from the online platform www.twitter.com, quering its public API by keyword (s) and hashtag(s). The sample size (number of users in the final dataset) was not decided a priori, but it is rather a by-product of the keyword selection
Data collection	- The Spanish electoral dataset was collected from the public Twitter API, using a selection of relevant keywords.<br>- The Catalan referendum dataset was extracted from the Twitter decahose (an enterprise API that delivers a 10% random sample of the realtime Twitter Firehose through a streaming connection), after filtering through a selection of relevant keywords and user accounts.<br>- Nepal Earthquake 2015, UEFA Football 2012, Hong Kong protests 2014 and Charlie Hebdo shooting 2015 were collected and kindly shared by Zubiaga A (see section Data above). Details on these datasets are published in "A longitudinal assessment of the persistence of Twitter datasets". Journal of the Association for Information Science and Technology, 69(8):974–984, 2018.
Timing	Spanish electoral period 2019: April 12 - May 6<br>Nepal Earthquake 2015: May 8 - May 14<br>Catalan referendum 2014: Sep 2 - Nov 12<br>UEFA Football 2012: June 19 - July 4<br>Hong Kong protests 2014: September 27 - October 7<br>Charlie Hebdo shooting 2015: Jan 8 - Jan 9
Data exclusions	No data were excluded from the analyses
Non-participation	No participants dropped out
Randomization	Participants were not allocated into experimental groups

Reporting for specific materials, systems and methods

We require information from authors about some types of materials, experimental systems and methods used in many studies. Here, indicate whether each material, system or method listed is relevant to your study. If you are not sure if a list item applies to your research, read the appropriate section before selecting a response.

Materials & experimental systems

n/a	Involved in the study	
☒	☐	Antibodies
☒	☐	Eukaryotic cell lines
☒	☐	Palaeontology and archaeology
☒	☐	Animals and other organisms
☒	☐	Human research participants
☒	☐	Clinical data
☒	☐	Dual use research of concern

Methods

n/a	Involved in the study	
☒	☐	ChIP-seq
☒	☐	Flow cytometry
☒	☐	MRI-based neuroimaging
